# A prospective randomised controlled trial investigating household SARS-CoV-2 transmission in a densely populated community in Cape Town, South Africa – the transmission of COVID-19 in crowded environments (TRACE) study

**DOI:** 10.1186/s12889-024-19462-1

**Published:** 2024-07-17

**Authors:** Philip Smith, Francesca Little, Sabine Hermans, Mary-Ann Davies, Robin Wood, Catherine Orrell, Carey Pike, Fatima Peters, Audry Dube, Daniella Georgeu-Pepper, Robyn Curran, Lara Fairall, Linda-Gail Bekker

**Affiliations:** 1 The Desmond Tutu HIV Centre, University of Cape Town, Cape Town, South Africa; 2 Department of Statistical Sciences, University of Cape Town, Cape Town, South Africa; 3 Amsterdam UMC, Department of Global Health, University of Amsterdam, Amsterdam, The Netherlands; 4 Centre for Tropical Medicine and Travel Medicine, Department of Infectious Diseases, Amsterdam UMC, Amsterdam Institute for Immunology and Infectious Diseases, University of Amsterdam, Amsterdam Public Health – Global Health, Amsterdam, The Netherlands; 5 Center for Infectious Diseases Epidemiology and Research, University of Cape Town, Cape Town, South Africa; 6 Western Cape Department of Health, Cape Town, South Africa; 7 Knowledge Translation Unit, University of Cape Town, Cape Town, South Africa

**Keywords:** SARS-CoV-2, COVID-19, Household transmission, Community health workers, Infection mitigation

## Abstract

**Background:**

South Africa’s first SARS-CoV-2 case was identified 5th March 2020 and national lockdown followed March 26th. Households are an important location for secondary SARS-CoV-2 infection. Physical distancing and sanitation – infection mitigation recommended by the World Health Organization (WHO) at the time – are difficult to implement in limited-resource settings because of overcrowded living conditions.

**Methods:**

This study (ClinicalTrials.gov NCT05119348) was conducted from August 2020 to September 2021 in two densely populated, low socioeconomic Cape Town community sub-districts. New COVID-19 index cases (ICs) identified at public clinics were randomised to an infection mitigation intervention (STOPCOV) delivered by lay community health workers (CHWs) or standard of care group. STOPCOV mitigation measures included one initial household assessment conducted by a CHW in which face masks, sanitiser, bleach and written information on managing and preventing spread were provided. This was followed by regular telephonic follow-up from CHWs. SARS-CoV-2 PCR and IgM/IgG serology was performed at baseline, weeks 1, 2, 3 and 4 of follow-up.

**Results:**

The study randomised 81 ICs with 245 HHCs. At baseline, no HHCs in the control and 7 (5%) in the intervention group had prevalent SARS-CoV-2. The secondary infection rate (SIR) based on SARS-CoV-2 PCR testing was 1.9% (*n* = 2) in control and 2.9% (*n* = 4) in intervention HHCs (*p* = 0.598). At baseline, SARS-CoV-2 antibodies were present in 15% (16/108) of control and 38% (52/137) of intervention participants. At study end incidence was 8.3% (9/108) and 8.03% (11/137) in the intervention and control groups respectively. Antibodies were present in 23% (25/108) of control HHCs over the course of the study vs. 46% (63/137) in the intervention arm. CHWs made twelve clinic and 47 food parcel referrals for individuals in intervention households in need.

**Discussion:**

Participants had significant exposure to SARS-CoV-2 infections prior to the study. In this setting, household transmission mitigation was ineffective. However, CHWs may have facilitated other important healthcare and social referrals.

## Introduction

South Africa’s first SARS-CoV-2 infection was detected on 5th March 2020 [1] and a national state of emergency was declared and nation-wide lockdown followed on March 26th in an effort to contain the spread of infection [2–4]. The lockdown prohibited all but essential services and severely limited all movement in and out of communities.

A large proportion of the 60 million people who make up the South African population live in low income, high density communities in peri-urban and newly urbanised locations [4, 5]. Physical distancing and sanitation – non-pharmaceutical interventions (NPIs) to mitigate infections recommended by the World Health Organization (WHO) at the time – were difficult to implement in limited-resource settings because of overcrowded living conditions [6, 7]. For example, many people did not have access to water within their dwelling and stood in queues to access water, food, or government grants.

The TRACE study (Transmission of COVID-19 in Crowded Environments) was a randomised controlled trial designed to measure the impact of an infection mitigation package on the frequency and timing of transmission of SARS-CoV-2 to household contacts in one such high density, low socioeconomic community in Cape Town South Africa. The package, called STOPCOV, was informed by WHO recommendations at the time [6]. STOPCOV was administered by trained lay community healthcare workers (CHWs). The primary objective was to investigate the transmission of SARS-CoV-2 to household contacts. The second objective was to investigate the effect of an infection mitigation package on household transmission of SARS-CoV-2.

## Methods

Setting and sample – The study was conducted in the Klipfontein Mitchells Plain (KMP) health sub-district in Cape Town, South Africa. KMP is a resource-limited, densely populated, high HIV/TB disease burden area in Cape Town [8]. The sub-district consists of a number of suburbs which house a population of approximately 1 million [9].

**Fig. 1:**
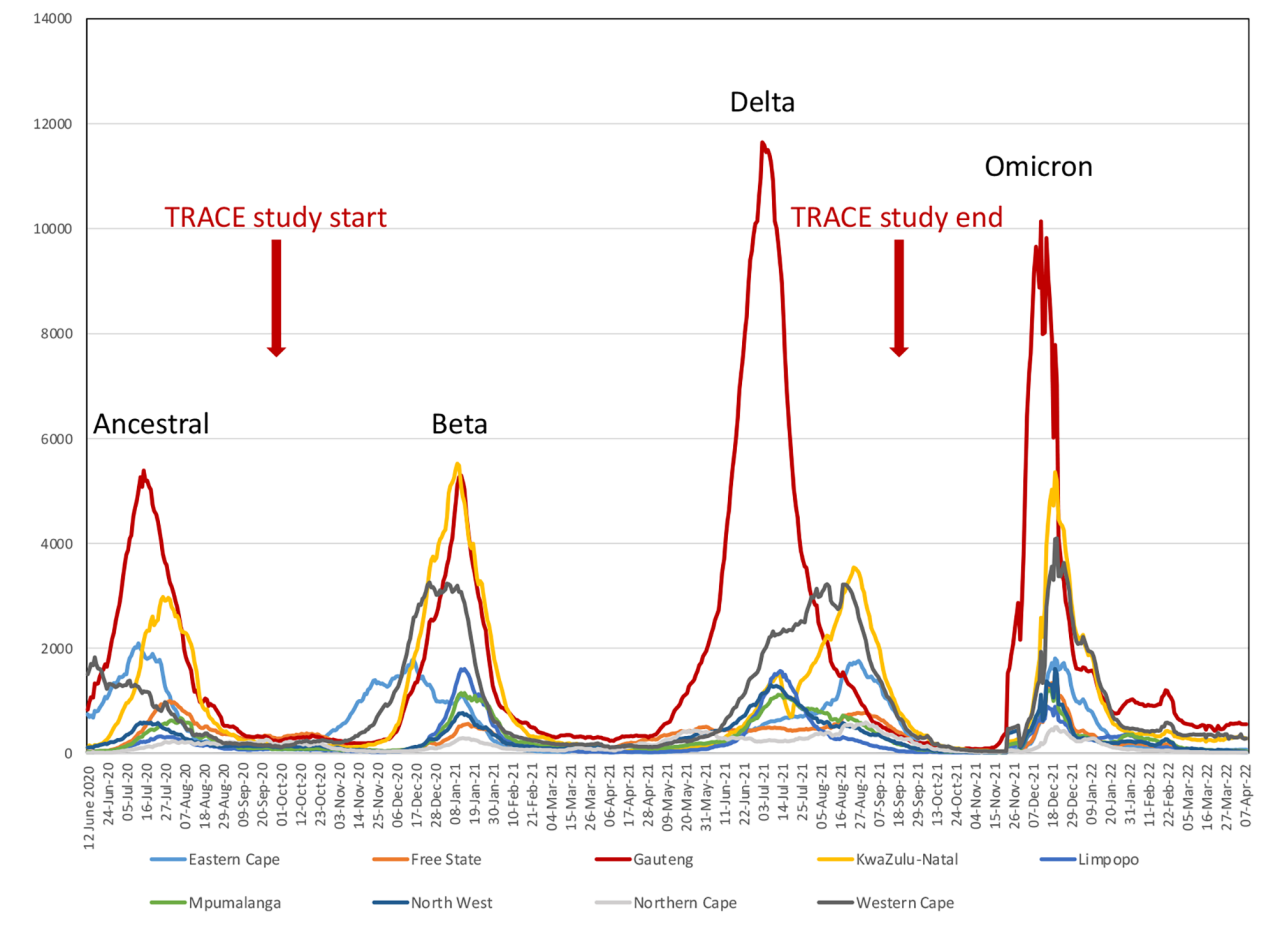
Seven day moving average of SARS-CoV-2 diagnosed cases by wave of infections in South Africa [12]

The study was conducted after South Africa’s COVID-19 first (ancestral variant) wave (23 August – 14 November 2020) and included the second (beta variant) (15 November 2020–6 February 2021) and third (delta variant) COVID-19 waves (28 March – 24 September 2021) [10] (Fig. 1). South Africa initiated a phased vaccine rollout for the general population in June 2021 starting with adults over 60 years, progressing to younger populations [11].

Newly diagnosed COVID-19 ICs were identified using PCR (polymerase chain reaction) testing at local public sector clinics in the district. Clinics notified their cases to the health district office, which then forwarded the contact information to the TRACE team. The study team was part of the sub-district’s contact tracing team, who contacted the individual and discussed possible participation in the study. ICs were included in the study if they were referred from a clinic with a positive SARS-CoV-2 test result, were 18 years and older, and were able to give consent. HHCs were included in the study if were living in the household of the IC, they were 12 years and older, a guardian had given consent, and were able to give assent. Participants were not excluded if they chose to isolate at a Department of Health facility. ICs were excluded if there were no HHCs. HHCs were excluded if they were under 6 years old.

Design – The study was a type 2 hybrid effectiveness-implementation [13] cluster randomised controlled trial with longitudinal follow-up of SARS-CoV-2 infection in 120 households with newly diagnosed positive ICs. This paper reports on the cluster randomised trial which was designed to measure the impact of an infection mitigation package on SARS-CoV-2 household transmission. Type 2 hybrid effectiveness-implementation was designed to examine the effectiveness of an intervention in real world settings to enhance adoption and sustainability, which will be reported elsewhere. Based on an exponential test for comparing hazards, and assuming 120 index cases (ICs) with 6 household contacts (HHCs) each, an intraclass correlation coefficient of 0.9, and a baseline R0 of 2.5 (resulting in an incidence of 0.208 among 720 subjects), the study had 80% power to detect a 40% decrease in incidence.

The study data analyst (FL) randomized households (*n* = 60 per condition) in blocks of four to STOPCOV, an intensified COVID-19 infection mitigation intervention administered by CHWs versus standard messaging provided by the Western Cape Department of Health (*n* = 60). Conducting randomization in blocks of four in an RCT helped ensure balanced group sizes across treatment and control arms, enhanced the statistical validity and reliability of the study, and maintained the blinding of researchers [14]. The study team conducting analyses were blinded to intervention assignment.

Based on the reduced sample size of 245 (137 in intervention group, 108 in control group), with average household size of 3, the power to detect the 40% decrease using the same assumptions as above reduced to 54%.

Procedure – Both groups received a standard message with their test result and received telephonic follow-up from the fieldworker to introduce the study and obtain locator information. Study fieldworkers allocated ICs and their households to their study arm after the initial telephonic contact. The ICs and their HHCs were enrolled after providing individual informed consent. The number of people living in the household was determined by how many people lived on the premises, including people who lived on the property but did not live in the main household (non-conventional household), or who lived on the premises periodically (household bubble), such as people who stayed only on weekends or during the week due to school or working arrangements. A baseline demographic, household characteristics questionnaire was administered by a fieldworker. For house visits, fieldworkers wore surgical gloves, aprons, and N95 facemasks.

SARS-CoV-2 screening (PCR testing using the TaqPath COVID-19 CE/IVD RT-PCR and IgM/IgG serology testing using the semi-quantitative Abbot Architect platform – semi-quantitative serology testing detects the presence and an estimate level of SARS-CoV-2 antibodies, however the test does not give a precise concentration) was performed by trained staff with ICs and their HHCs at baseline, weeks 1, 2, 3 and 4 of follow-up or until they received a positive test result. Specifically, the study conducted SARS-CoV-2 PCR testing on nasopharyngeal swab specimens taken from HHCs and blood draws (antibody serology) taken from ICs and HHCs. ICs did not receive repeat PCR testing since they had received a positive PCR test from the clinic before enrolment.

Intervention – The STOPCOV support comprised an initial household assessment to assess needs and offer isolation referral to a Department of Health facility. The CHWs also offered information about symptom management, infection control measures, assistance with adapting the measures to their homes, provision of basic supplies (masks, hand sanitiser, and bleach), and written materials at appropriate literacy levels and in the local language. The same lay CHWs provided daily follow-up of household members for the first two weeks and three times a week during weeks 3–4, and distributed pre-prepared daily text messages. CHWs were also on hand to refer for more urgent care, isolation or quarantine facilities, food parcels and linkage to clinics for other healthcare if appropriate. Control households did not receive additional support above the routine SARS-CoV-2 testing and encouragement to quarantine.

Analysis – Analyses were conducted using STATA 14 (Stata Corporation, College Station, TX). We calculated the proportion of SARS-CoV-2 transmission from ICs in each household over a 4-week period. Incidence was calculated as proportions of sero-negative people who turned sero-positive, without regards for person-time and compared using a design-based F-statistic. We tested the impact of the infection mitigation intervention by comparing the incidence in households randomised to intervention versus standard of care.

Ethics – The study was reviewed and approved by the University of Cape Town Health Science Research Ethics Committee (Reference 284/2020). Approval to conduct research was provided by the Western Cape Provincial Department of Health and supported by the Community Advisory Board. Informed consent was obtained from all participants for enrolling in the study. The trial was registered and the protocol was uploaded on ClinicalTrials.gov (NCT05119348, registered date: 15/11/2021).

## Results

**Fig. 2:**
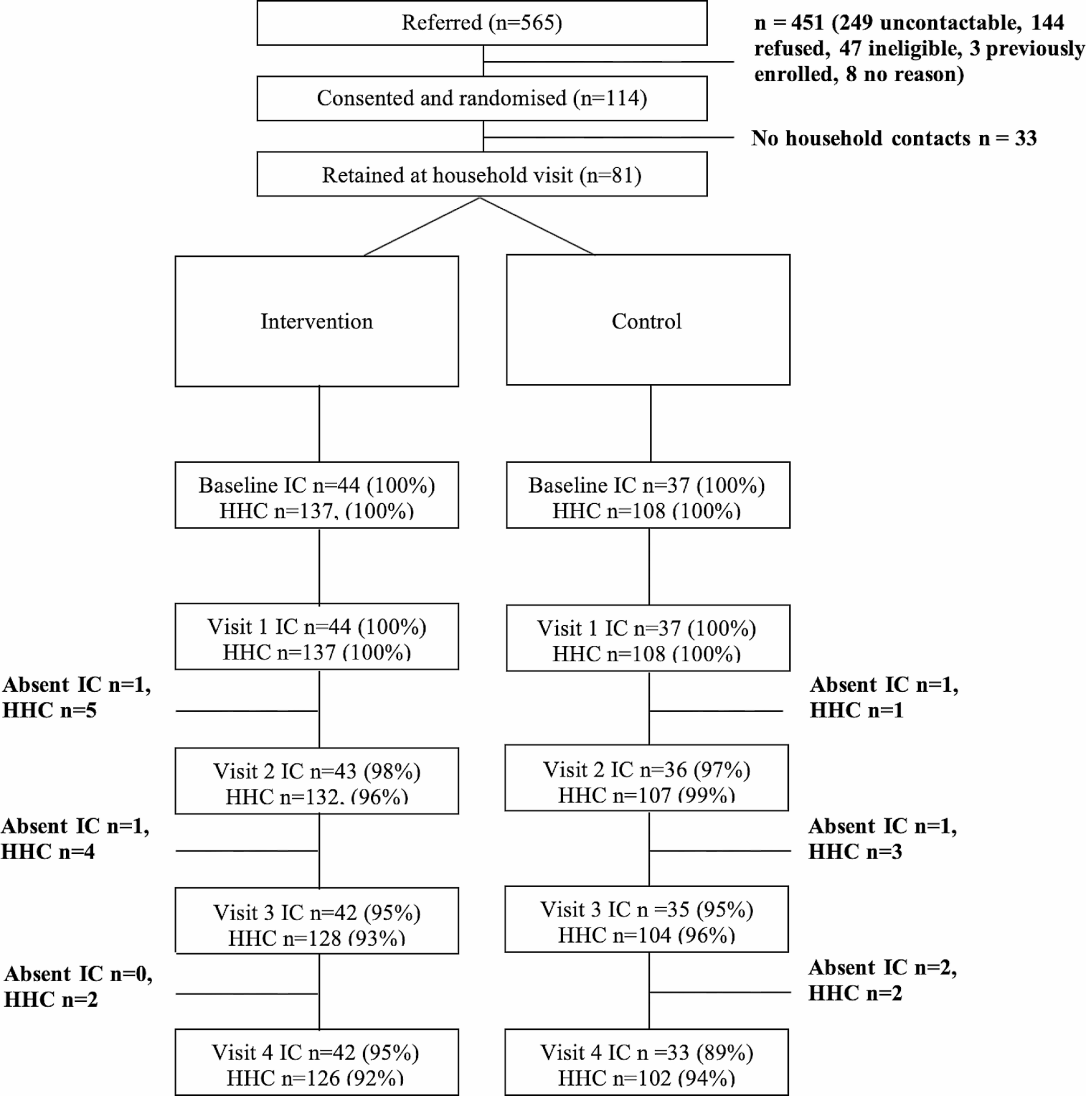
Consort IC = index case, HHC = household contact

Between September 2020 and September 2021, 565 (Fig. 2) patients were referred from local clinics via the district health office to the study team. Referring clinics informed patients of their test results between one and four days after testing. Of those referred for consideration in the study, 451 were not included for the following reasons (Fig. 2): 249 had missing or incorrect contact information, 144 refused participation, 47 were ineligible (17 late referrals, 13 lived outside the study area, 8 had no HHCs, 4 referrals were already HHCs of an enrolled IC, 2 were unavailable at visit, 1 was not tested, 1 received a negative SARS-CoV-2 test result, 1 underage 8 year old), 3 were ICs from previously enrolled households, and 8 had no reason recorded. The study randomised 114 eligible ICs and their households. All 57 intervention households received an introductory visit by the CHWs. Two were excluded: one because the IC had died following diagnosis and the family were travelling to their rural home for the funeral, and another one because the IC had been immediately hospitalised following diagnosis with COVID-19 and TB and expected to spend a protracted time in hospital. The remaining 55 intervention households received follow-up calls (median 12 calls; IQR 8–11) and text messages (median 10 messages, IQR 10–12 messages). Total contact time by telephone varied markedly across households and by CHW but ranged from 6 min 15 s to 2 h 2 min and 28 s (median 21 min).

The fieldworker team that visited the households for sample collection and questionnaire completion found that several HHCs were unavailable or refused to participate in the study. Although all randomised ICs had stated that they had HHCs, 33 households were excluded because this was not the case upon visiting the house. Ultimately, 81 (71%) (37 in the control and 44 in the intervention) households with at least one HHC were enrolled, with 14 (38%) and 16 (36%) male ICs in the control and intervention respectively (Table 1).

There was a total of 245 HHCs (108 in the control, 137 in the intervention), where almost half (43%) were male (not all HHCs gave consent to participate). ICs were older in the intervention than the control (median age: control 39 years (IQR 31–58 years), intervention 52 years (IQR 41–66 years), *p* = 0.0159). Overall, there were 23 HHCs under 18 years enrolled (*n* = 13 in the control, *n* = 10 in the intervention). The univariate analysis indicated no statistically significant age difference between the control and intervention groups. There was no difference between HHC age between arms (mean age: control 40 years: IQR 26–50 years, intervention 38 years: IQR 25–49 years, *p* = 0.472). The average days to enrolment from testing was 5 days (range 2–14 days), which was not significantly different between arms.

**Table 1:** Baseline characteristics of index cases and their household contacts

	Control		Intervention		Total		*p*-value▴
	*n*	(%)	*n*	(%)	*n*	(%)	
**Index**	37		44		81		
Age in years, median (IQR)	39 (31–58)		52 (41–66)		47 (36–62)		0.0159
Male	14	(38%)	16	(36%)	30	(37%)	0.08922
Household							
Conventional	31	(84%)	34	(77%)	65	(80%)	0.5701
Two or more structures	3	(8%)	7	(16%)	10	(12%)	
Bubble - two or more locations	3	(8%)	3	(7%)	6	(8%)	
Dwelling type (Brick or shack)							0.2813
Brick	29/32	(91%)	33/34	(97%)	62/66	(94%)	
Built in toilet							0.0771
Yes	20/32	(63%)	28/34	(82%)	48/66	(73%)	
Mean HHCs per Index (IQR)	2.9	(1:3)	3.1	(1:4.5)	3	(1:4)	
**Household Contacts**	108		137		245		
Age in years, median (IQR)	41 (26–50)		35 (25–49)		37 (26–49)		0.4723
Sex male	48	(44%)	58	(42%)	106	(43%)	0.7724
Household							0.4768
Conventional	65	(60%)	89	(65%)	154	(63%)	
Two or more structures	17	(16%)	32	(23%)	49	(20%)	
Bubble - two or more locations	26	(24%)	16	(12%)	42	(17%)	
Dwelling type (Brick or shack)							0.1203
Brick	104/108	(96%)	121/136	(89%)	225/244	(92%)	
Built in toilet							0.4604
Yes	81/108	(75%)	91/136	(67%)	172/244	(70%)	

▴values provided for HHCs where transmission was measured over four weeks of follow-up

**Table 2:** HHCs SARS-CoV-2 PCR and anti-body serology results over 4-week follow-up HHCs SARS-CoV-2 PCR and anti-body serology (anti-nucleocapsid antibody testing) results over 4-week follow-up (intervention *N* = 137, control *N* = 108)

HHC	Baseline		Week 1		Week 2		Week 3		Week 4		Cumulative	
	*n*/*N*	(%)	*n*/*N*	(%)	*n*/*N*	(%)	*n*/*N*	(%)	*n*/*N*	(%)	*n*/*N*	(%)
PCR												
Control	0/137	0%	2/137	1%	0/132	0%	0/128	0%	0/126	0%	2/137	1%
Intervention	7/108	6%	1/108	1%	1/107	1%	1/104	1%	1/102	1%	11/108	11%
Ab serology												
Control	16/137	12%	3/137	2%	1/132	1%	1/128	1%	4/126	3%	25/137	18%
Intervention	52/108	48%	5/108	5%	2/107	2%	1/104	1%	3/102	3%	63/108	58%

The antibody and PCR test was not repeated after the participant tested positive, thus final column cumulative

Of the HHCs who were tested at baseline, SARS-CoV-2 antibodies were present in 12% of control and 48% of intervention participants (Table 2). At baseline, no HHCs in the control and 7 (6%) in the intervention received a positive SARS-CoV-2 PCR test result. At the end of 4-week follow-up, SARS-CoV-2 antibodies were detected in an additional 9 control arm and 11 intervention arm HHCs. Antibodies were detected in 18% of control and 58% of intervention HHCs over the course of the study.

The exponential proportional hazard regression analysis showed a relative hazard for seroconversion of 1.98 (*p* = 0.059) in the intervention group compared with the control group. The SIR based on SARS-CoV-2 PCR testing in HHCs was 2.06% (*n* = 2) in control households vs. 3.23% (*n* = 4) in intervention households (Table 3) over the four weeks of follow-up (*p* = 0.598). Additionally, for serology testing, the incidence was 9.00% (*n* = 9) in control households and 8.09% (*n* = 11) in intervention households over follow-up (*p* = 0.804). There was an overlap of two individuals who both seroconverted and tested PCR positive. Overall, 24 unique individuals either seroconverted after baseline blood collection or tested PCR positive.

**Table 3:** PCR and serology test result change from negative to positive

	*n*/*N*	Incidence	*P*
PCR			0.5975
Control	2/97	2.06	
Intervention	4/124	3.23	
Serology			0.8039
Control	9/100	9.00	
Intervention	11/136	8.09	

Lastly, in the intervention group, the CHWs made 12 clinic referrals for patients to re-engage with chronic care (*n* = 6 HIV or NCDs, *n* = 4 new or persistent COVID-19 symptoms, *n* = 1 TB screening, *n* = 1 antenatal care), and 47 food parcel referrals for households experiencing food insecurity with limited access to food. A CHW followed up with participants who were referred for food parcels to ensure delivery. Two HHCs were offered isolation referral but declined, stating that they preferred to stay with family during recovery.

## Discussion

This study confirms there was significant transmission of SARS-CoV-2 during the first wave in crowded low socioeconomic settings in Cape Town with antibodies detected in 38% of the intervention group and 15% of the control group at baseline. Rapid identification and isolation of new cases, with tracing and tracking of possible contacts in order to mitigate further spread through quarantine, physical distancing and other NPIs was a strategy employed early in the epidemic especially before vaccination was available [15].

The high level of antibodies in this study was similar to high antibody prevalence in blood donor [16] and residual routine care specimens from antenatal visit [17] studies at the time. Additionally, prevalence data for the end of wave three from Cape Town showed that there was high seroprevalence around 70% in poorer communities [18]. In September 2020, after the first wave had subsided and when this study commenced, antibodies to SARS-CoV-2 were detected in 1123 of 2791 (40%) individuals attending primary health care services in the Cape Town Metropolitan for antenatal care and HIV care [17]. A series of seroprevalence studies in different communities in the Western Cape confirmed high rates of infections, especially in poorer crowded communities following the first wave and the lockdown [18].

The evidence did not support the hypothesis that the STOPCOV intervention would reduce household transmission. Since SARS-CoV-2 is highly transmissible, is airborne and causes mild symptoms in most who are infected [19], an infection may go unnoticed in the household until a positive SARS-CoV-2 diagnosis is made. The high level of SARS-CoV-2 antibodies in HHCs at baseline suggests that there was prior infection in HHCs. We also know there was very high transmission of infection in these communities during the lockdown [18]. In comparison, findings in other household studies indicated a SARS-CoV-2 secondary infection rate to HHCs between 4% and 55% [20–23] and a large South African study indicated a HHC infection rate of 24%. Our serological findings in the control group (9 out of 100, incidence 9.00) and intervention group (11 out of 136, incidence 8.09) are relatively low in comparison. These results suggest a comparatively lower secondary infection rate in the current study compared to the reported rates in other studies.

Although contact tracing was a key primary public health intervention at the time, this study illustrates the logistical challenges in its implementation. Almost three quarters (71%) of ICs referred to the study team were unreachable by phone or refused to participate. Participants were only able to be enrolled an average of five days and up to two weeks after their SARS-CoV-2 PCR test was conducted, potentially resulting in household transmission occurring before infection mitigation could be initiated. Of the 55 intervention households, only 11 were visited by the CHW within 6 days of symptom onset of the IC, and the median was 9 days meaning that opportunity to mitigate spread had been missed. Delayed contact tracing, although still providing value in identifying household infections and managing illness [15], has limited benefit in reducing onward transmission within households in these contexts [24]. Utility and the timing of contact tracing strategies should be carefully considered in the timing and nature of the epidemic response [15, 25] since there is also the cost of public health resources and social stigma burden on the household [26].

Although the STOPCOV intervention was not associated with a difference in infection between the two groups, likely due to the lag between testing and enrolment, there was an indication that the intervention provided benefit. The intervention supported households navigating a COVID-19 diagnosis amidst everyday contextual realities, including accessing various resources, including medical care and social relief. This additional support was critical at the time where many routine and medical interventions for acute conditions were delayed during the lockdown [27]. Since clinics were closed for routine care and there was uncertainty about healthcare provision during the lockdown, there was a 22% decline in HIV testing and 26% decline in TB testing in 2020 compared with the previous year [28]. An important benefit of the STOPCOV intervention was that community health workers assisted with managing COVID-19 in the household and referred those in need of additional care or other critical support, such as food security, to relevant services. Initial household visits and routine calls from trained CHWs may improve household support and access to information. These interactions between CHWs and patients may mitigate health-seeking delays to minimise adverse outcomes through regular contact with ICs and their households.

There were limitations in conducting this study. Only 46% (*n* = 114 of 316) of contactable newly identified COVID-19 cases agreed to participate. This may indicate that follow-up was associated with stigma [29], or that participants associated repeated follow-up to be burdensome, undesirable [30] or intrusive [31]. Additionally, participants may have had concerns about being referred to a quarantine field hospital away from family [32]. On telephone screening, only ICs with known HHCs could be considered eligible. However, not all participants were confirmed to have at least one HHC when the study team arrived, which meant the household was not included in the study. Participants who consented but did not have HHCs were excluded, leading to an unbalanced allocation with more participants in the intervention group. The reduced sample size was a limitation which posed a challenge to achieving adequate statistical power, which in turn impacted the statistical analysis. This constraint may have impacted the sensitivity analysis, resulting in outliers or extraneous variables disproportionately influencing results and robustness. To mitigate this, an adequate sample size would provide greater statistical power and improve the reliability and generalizability of the findings. Nevertheless, we believe the findings presented provide valuable insights within the scope of the available data. The study groups were also unbalanced at baseline with more HHCs with both a positive SARS-CoV-2 PCR test result and reactive antibody result in the intervention group. It is difficult to assess the impact of this imbalance on the study outcomes. Another limitation of the study is that we did not establish a true index case. The identification of an IC is an important aspect of contact tracing; however, we did not employ methods to identify undiagnosed index cases in the household. While it may be worth considering methods of identifying the true index case, the identification of any case may still be beneficial to manage infection and mitigation in the household.

Another limitation was the lengthy period between a positive PCR SARS-CoV-2 test in a potential IC by the clinics and referral to study staff. During an infection wave, the numbers of positive cases meant delays both in laboratory testing and clinic processes of patient notification. Clinics routinely sent results to participants up to a week after testing. Within the period of testing through to receiving test results, even with isolation of the IC, HHCs may have acquired SARS-CoV-2.

## Conclusion

In this study household contact tracing and targeted use of NPIs through trained CHWs was not associated with a reduction in SARS-CoV-2 transmission. Household transmission of SARS-CoV-2 likely occurred before the IC was identified, which may have been impacted by referral delays. A significant portion of South Africa’s population resides in densely populated, low-income communities situated in peri-urban and recently urbanized areas, which have significantly higher social mixing [5], which may place these communities at higher risk of the rapid spread of respiratory disease and epidemics. Consequently, contact tracing may have limited application for mitigating infection in this setting of a highly crowded environment. Other context specific interventions may be better suited to managing pandemics in limited resources settings, such as South Africa. In these settings, community-based disease management with community-based health and social support, coupled with communication in at-risk households may provide crucial support for those most in need.

## Data Availability

The datasets generated and/or analysed during the current study are available in the zivahub.uct.ac.za repository, https://zivahub.uct.ac.za/articles/dataset/TRACE_Transmission_of_COVID19_in_Crowded_Environments_all_study_data/20473056.
